# Nitroxides as Antioxidants and Anticancer Drugs

**DOI:** 10.3390/ijms18112490

**Published:** 2017-11-22

**Authors:** Marcin Lewandowski, Krzysztof Gwozdzinski

**Affiliations:** Department of Molecular Biophysics, Faculty of Biology and Environmental Protection, University of Lodz, 90-136 Lodz, Poland; marcin.lewandowski@biol.uni.lodz.pl

**Keywords:** nitroxide, antioxidant properties, nitroxide as drugs, spin labelled drug analogues, oxidative stress

## Abstract

Nitroxides are stable free radicals that contain a nitroxyl group with an unpaired electron. In this paper, we present the properties and application of nitroxides as antioxidants and anticancer drugs. The mostly used nitroxides in biology and medicine are a group of heterocyclic nitroxide derivatives of piperidine, pyrroline and pyrrolidine. The antioxidant action of nitroxides is associated with their redox cycle. Nitroxides, unlike other antioxidants, are characterized by a catalytic mechanism of action associated with a single electron oxidation and reduction reaction. In biological conditions, they mimic superoxide dismutase (SOD), modulate hemoprotein’s catalase-like activity, scavenge reactive free radicals, inhibit the Fenton and Haber-Weiss reactions and suppress the oxidation of biological materials (peptides, proteins, lipids, etc.). The use of nitroxides as antioxidants against oxidative stress induced by anticancer drugs has also been investigated. The application of nitroxides and their derivatives as anticancer drugs is discussed in the contexts of breast, hepatic, lung, ovarian, lymphatic and thyroid cancers under in vivo and in vitro experiments. In this article, we focus on new natural spin-labelled derivatives such as camptothecin, rotenone, combretastatin, podophyllotoxin and others. The applications of nitroxides in the aging process, cardiovascular disease and pathological conditions were also discussed.

## 1. Introduction

Nitroxides belong to a group of stable organic radicals, containing the nitroxyl group >N–O^•^ with an unpaired electron [1]. They have a low molecular weight, are non-toxic, do not elicit immunogenic effects on cells and easily diffuse through cell membranes [2]. Their biological activity as antioxidants is related to the regulation of redox state in the cells. Nitroxides can undergo one-electron oxidation or reduction reactions. Their antioxidant activity is related to the direct scavenging of free radicals, transition metal ion oxidation in the reduction of hydrogen peroxide in the Fenton reaction and other peroxides and catalyzing Haber-Weiss reactions. In addition, nitroxides exhibit superoxide dismutase (SOD)-like activity, modulate its catalase-like activity and ferroxidase-like activity, and are the inhibitors of free radical reactions such as lipid peroxidation [3,4]. In general, nitroxides inhibit oxidative stress, although under certain conditions they may also lead to its intensification, for example, in tumour cells. This situation occurs at high nitroxide concentrations that can release iron ions that participate in the Fenton and Haber-Weiss reactions [2,5,6].

Unlike other antioxidants, they are characterised by a catalytic mechanism of action associated with a single-electron redox cycle. Their reduction results in the generation of hydroxylamine and oxidation in oxoammonium ion; meanwhile both reactions are reversible [7]. Hydroxylamine also exhibits antioxidant properties because it is easily oxidised to nitroxide. As mentioned above, the nitroxides devoid of electrical charge can easily diffuse through the cell membranes, thus they can also inactivate the reactive oxygen species formed in the cells and modulate the concentration of intracellular nitric oxide [8].

A summary of the antioxidant properties of nitroxide has recently been published considering Tempol—the most commonly studied nitroxide [9].

Some earlier studies have used nitroxides in electron paramagnetic resonance as probes and spin labels. However, their properties can also be used as contrast enhancing agents in MRI (magnetic resonance imaging) and as photoprotective and radioprotective substances [10]. As contrast enhancing agents, they have an ability to detect subtle changes in redox equilibrium in the tumor tissue and their application allows distinguishing the normal and pathological states of tissues. In addition to the aforementioned properties, nitroxides also have other broad range of bioactivities, such as anti-inflammatory [11], neuroprotective effect [12], antinociceptive effect [13] and antitumor activity [14].

Owing to their chemical and physical properties, their metabolism and detailed mechanism have been described in detail in other papers [1,15,16,17]. In this review, we present their practical applications as antioxidants and drugs in the treatment of cancer as well as neutralising the oxidative stress induced by anticancer drugs used in standard chemotherapy. We have also discussed the application of new natural spin-labelled compounds such as camptothecin, rotenone, combretastatin, podophyllotoxin and others. Nitroxide roles in inhibiting inflammation, angiogenesis and oxidative stress have been also reported.

## 2. Properties of Nitroxides

**Figure 1:**
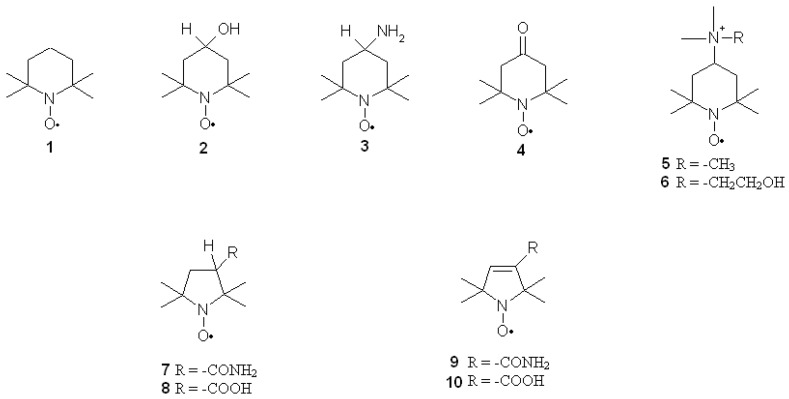
Most commonly used piperidine, pyrroline and pyrrolidine nitroxides: (**1**) Tempo, (**2**) Tempamine, (**3**) Tempone, (**4**) Tempol, (**5**) CAT-1, (**6**) Tempocholine, (**7**) Pirolid, (**8**) carboxy-Pirolid, (**9**) Pirolin (PL) and (**10**) carboxy-Pirolin.

Nitroxides are a group of aliphatic, aromatic, bicyclic or heterocyclic stable radicals. The most commonly applied and examined heterocyclic nitroxides are the derivatives of piperidine, pyrroline, pyrrolidine, oxazolidine, imidazoline and imidazolidine. The stability of nitroxides of this group is due to the lack of hydrogen atoms at alpha carbon atom (neighboring >N–O^•^ group). Most of the derivatives of piperidine, pyrroline and pyrrolidine are water soluble, which facilitates their application on biological systems. On the other hand, the oxazolidine derivatives are often fatty acids, steroids or lipids, which can be used as spin labels as they are insoluble in water. Unless they are charged, nitroxides easily penetrate through cell membranes. Moreover, nitroxides are non-toxic and non-immunogenic for normal cells. The structures of the most commonly used nitroxides are presented in Figure 1.

**Figure 2:**
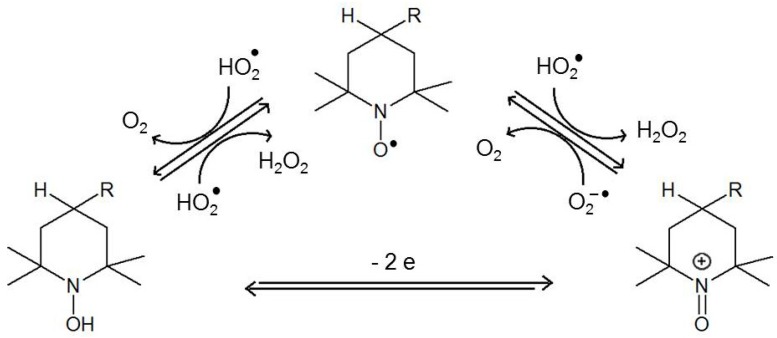
Redox cycle of nitroxides and their SOD-like activity.

Nitroxides possess antioxidative properties owing to their ability to undergo redox cycles. They are easily reduced to hydroxylamines and oxidized to oxoammonium salts (direct conversions between hydroxylamines and oxoammonium salts are possible in two-electron reactions (Figure 2) [18].

Under certain conditions, nitroxides can be reduced to their corresponding secondary amines. The reducing agents include sulphides, thiols and zinc [19,20,21]. Nitroxides metabolize superoxide ion radicals and their protonated forms (hydroperoxyl radical) into hydrogen peroxide and oxygen, thus acting as SOD mimetics (Figure 2) [18,22,23,24,25,26].

Nitroxides also display pro-oxidant properties, similar to other antioxidants as flavonoids and vitamins. In cells, nitroxides are mainly reduced by ascorbic acid with the help of thiols [27]. Erythrocytes incubated with nitroxides are characterised by thiol depletion, especially glutathione (GSH) [28,29]. The presence of oxygen is also crucial for nitroxide reduction, as it is faster in anaerobic conditions [30]. The derivatives of piperidine are reduced faster than pyrrolines and pyrrolidines [31,32,33] and the non-charged derivatives of piperidine are reduced in cells faster than charged ones [33,34]. A study of ours [30,32] showed that nitroxides are not metabolised in erythrocytes, which was further confirmed in tissues [35]. The reduction rate of piperidines also depends on the type of substituent at position 4 of the heterocyclic ring [30]. For instance, the reduction rate of piperidine nitroxides is as follows: Tempamine > Tempone > Tempol > Tempocholine (Figure 1). The reduction rate of pyrrolines and pyrrolidines is as follows: Pirolid > Pirolin > carboxy-Pirolid > carboxy-Pirolin [32,33]. Nitroxides also display catalase-like activity and inactivate hydrogen peroxide by oxoammonium cation [36,37,38,39] or hydroxylamine [40]. Being free radicals, nitroxides take part in the recombination reactions; they inactivate free radicals that initiate oxidation of lipids and proteins. These reactions can also be inhibited by nitroxides reacting with lipid radicals, interrupting lipid peroxidation [41,42,43]. As previously mentioned, oxoammonium cations can be reduced to hydroxylamines by ascorbic acid. This reaction yields ascorbyl radicals, which undergo dismutation to produce ascorbate and dehydroxyascorbate. It is also catalysed by nitroxides [44]. Nitroxides inhibit lipid peroxidation induced by the Fenton reaction in rat heart, liver and kidney homogenates and reduce rat erythrocyte haemolysis induced by hydrogen peroxide [45]. Nitroxides have shown to scavenge ROS in the following order: hydroxyl radicals > hydrogen peroxide > superoxide. TEMPOL (4-hydroxy-2,2,6,6 tetramethylpiperidine-1-oxyl) was found to effectively scavenge or suppress formation of hydroxyl radicals inside Cu, Zn-SOD. It also inactivates singlet oxygen, peroxyl and alkoxyl radicals, nitrogen dioxide [41,46,47] and strong oxidizing and nitrating agent peroxynitrite [48,49,50,51]. As free radicals, nitroxides are also scavengers of carbon-centered radicals [52]. Nitroxides oxidise transient metal ions that take part in the Fenton and Haber-Weiss reactions, preventing biological material from oxidative damage [30,53,54,55] and exhibit ferroxidase-like activity [21].

## 3. Nitroxides in Cancer Therapy

In chemotherapy, various antineoplastic drugs aiming to destroy or at least inhibit the tumour growth are used. Their effect depends on the individual malignancy of a tumour as well as the attainment of the desired concentration of the drug in blood plasma and the target organ. In last few years, the effectiveness of the drugs commonly used in cancer therapy has been improved considerably. The research is being conducted in order to find compounds with greater bioactivity and toxicity against the tumour cells but being safe to the normal cells. Nitroxides meet with these conditions as they are not toxic to host cells and exhibit toxicity only to tumour cells. The addition of nitroxide to an anticancer drug molecule gives the drug molecule new properties. A spin-labelled anticancer drug often exhibits several times more potent effects on the tumour tissue than the unmodified drug.

### 3.1. Breast Cancer

It has been established that chemotherapeutics used in breast cancer therapy, for example, fluvastatin (**25**), are toxic not only to cancer cells but also to normal cells. This problem may be solved by co-administration of nitroxides, for instance, Mito-CP_11_ (**11**), whose specific accumulation in mitochondria occurs due to the presence of a charged group that facilitates penetration through the cell membrane. Cheng et al. [56] showed that after 48 h of administrating 0.5 μM Mito-CP_11_, the viability of MCF-7 (breast cancer cell line) cells was dropped by approximately 30% while there was no impact on the viability of MCF-10A normal mammary epithelial cells. The beneficial effects were also observed for concomitant administration of Mito-CP_11_ with fluvastatin (0.5 μM and 1.0 μM, respectively, for 48 h) against MCF-7 cells, resulting in around 80% inhibition of cancer cell colony formation, while when the compound was applied individually, it caused only 25% inhibition. Comparable results have also been observed for other breast cancer cell line, namely MDA-MB-231. Some tests (colony formation assay, MTT test, [^3^H]-thymidine uptake) also led to the conclusion that Mito-CP_11_ enhances the cytotoxicity of fluvastatin against breast cancer cells, while simultaneously being low-toxic to the normal cells. This differential activity can be explained by the specific inhibition of NF-κB activity, which is a characteristic of MCF-7 breast cancer cells line but not of a normal cell [56].

**Figure 3:**
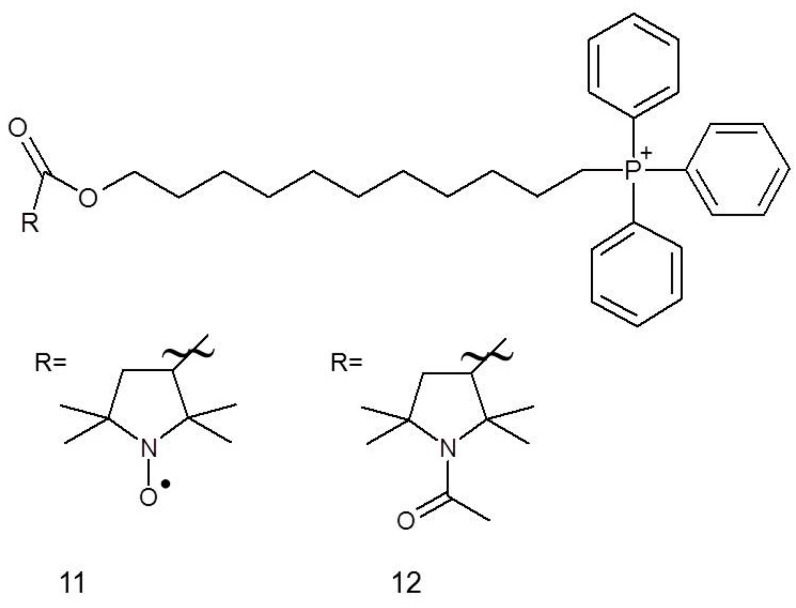
Nitroxides and their nonparamagnetic derivatives used as anticancer drugs: (**11**, **12**) Mito-Cp_11_ and Mito-Cp_11_-Ac.

It has recently been reported that both Mito-CP (nitroxide) and Mito-CP-Ac (non-paramagnetic acetamide derivative) can potently inhibit tumour cell proliferation (Figure 3). Both compounds altered mitochondrial and glycolytic functions; the intracellular citrate levels caused a depletion of intracellular ATP and induced apoptosis in both breast cancer cell lines, MCF-7 and MDA-MB-231 [57].

The application of nitroxides in breast cancer therapy is not only limited to their role in cytostatics; they may also be used in combination with other drugs to alleviate their side effects. For instance, nitroxides may reduce cardio-, hepato- and nephrotoxicity of doxorubicin and taxanes. These side effects are most probably induced by oxidative stress and may result in the reduction of drug doses and improve low therapeutic efficacy. A solution of nitroxide Pirolin (**9**) exerts its effect by antioxidative properties and ability to oxidise Fe(II) in non-targeted tissues [58]. Pirolin protected blood plasma in a mammary tumour bearing rat model administered with a combination of doxorubicin and docetaxel. Nitroxides can protect proteins against oxidation (the carbonyl group formation in proteins as biomarker of oxidative damage of proteins) having no influence on thiol groups. Interestingly, Pirolin showed both pro- and antioxidative effect on lipid peroxidation (a concomitant increase of hydroperoxides and reduction of TBARS content), which can be an evidence of inhibition of fragmentation of hydroperoxides and reduction of the concentration of products reacting with thiobarbituric acid (TBA). The peroxidation process can be stopped after hydroperoxide formation but both Fe(II) and Fe(III) can reinitiate degradation of hydroperoxide. However, the toxicity of doxorubicin and docetaxel is limited mainly to heart, liver and kidneys; therefore, the protective abilities of Pirolin might be different in these organs [58].

Recently, we have shown the attenuation of tumour progression by Pirolin nitroxide. The median number of tumours per rat and its volume, at the end of the study, were considerably smaller in Pirolin nitroxide-treated groups than the control group that was given only Pirolin (**9**). No negative changes were observed in the heart tissue of the animals after Pirolin treatment [59].

**Figure 4:**
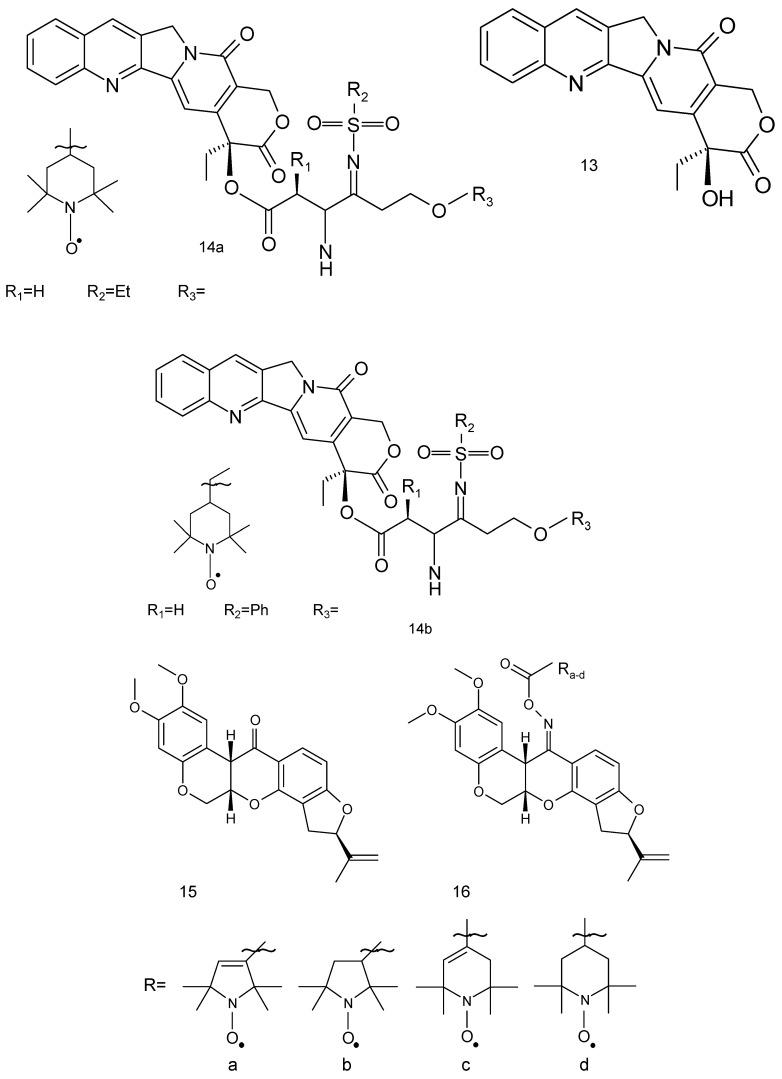
The natural and spin-labelled derivatives used against cancer cell lines: (**13**) camptothecin, (**14**) spin-labelled camptothecin, (**15**) rotenone and (**16**) the spin-labelled derivatives of rotenone.

The derivatives of camptothecin constitute a group of antitumor agents. Camptothecin (**13**) is a pentacyclic alkaloid, isolated from *Camptotheca acuminate* (Figure 4) [60]. These compounds showed good antitumor activity against a broad spectrum of tumour cell lines and clinical utility in the treatment of human malignancies such as colorectal, lung (small-cell lung cancer) and ovarian tumours. Two camptothecin derivatives, topotecan and irinotecan, are widely used for the treatment of human solid tumours. Camptothecins target topoisomerase I and stabilise the binding of topoisomerase I to DNA.

Eighteen spin-labelled analogues of camptothecin were tested against MDA-MB-231 cells. Two compounds (**14a**, **14b**) exhibited high antiproliferative activities against four tested tumour cell lines (IC_50_ 0.080 μM and 0.091 μM, respectively) [61].

Another compound of plant origin is rotenone flavonoid (Rot) (**15**). Its derivatives are present in *Derris elliptica*, *Lonchocarpus utilis* and other Legumes (Figure 4). Rot interferes with the electron transport chain in mitochondria and its toxic effects could be attributed to the inhibition of mitochondrial NADH dehydrogenase in complex I [62]. This flavonoid derivative exhibits anticancer activity through the generation of ROS and induction of apoptosis in various cancer cells and displays a strong antiproliferative effect against human breast cancer MCF-7 cells. Rot leads to chromatin condensation and cleavage of polymerase (PARP), which culminates in apoptosis in MCF-7 cells. Additionally, it causes the activation of the c-jun N-terminal kinase (JNK) and p38 mitogen-activated protein kinases (MAPKs) [63]. However, its spin-labelled analogue (**16**) was also tested in A-549, DU-145, Kb and Kbvin cancer cell lines.

### 3.2. Hepatic Cancer

The studies performed on HepG2 cell line showed that Mito-CP_11_ is also effective in liver cancer therapy [64]. On the basis of the fact that cancer cells are characterised by enhanced glycolysis, Mito-CP_11_ was co-administered with 2-deoxyglucose (2-DG) known as a glycolysis disrupting compound. When nitroxide and 2-DG were administered individually, no effect on the normal and cancer cell viability was noted, whereas 2 μM Mito-CP_11_ and 1 mM 2-DG (24 h incubation) caused 50% reduction in HepG2 viability without no influence on normal hepatocytes. An incubation period of 6 h for the cells with a combination of drugs (concentrations as above) disrupted the energy production processes resulting in 60% reduction of ATP content in the cancerous cells. The cytotoxicity of the combination of Mito-CP_11_ and 2-DG was confirmed by the induction of apoptosis, increase in caspases 3 and 7 activity, and altered expression of anti- and proapoptotic proteins. The results showed a synergistic effect of Mito-CP_11_ and 2-DG [64].

**Figure 5:**
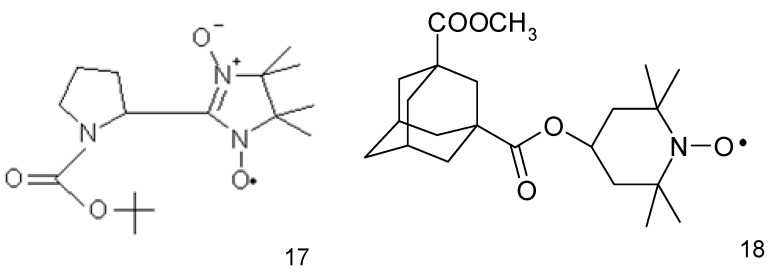
Nitroxides used as anticancer drugs: (**17**) L-NNP and (**18**) adamantyl nitroxide derivative.

The anticancer activity of nitroxides towards HepG2 liver cancer cell line was also demonstrated by Guo et al. (2012) [65] while investigating the imidazoline nitroxide L-NNP (**17**) (Figure 5). It was found to be selective for liver cancer cells, while being non-toxic to normal hepatocytes (IC_50_ 5.6 and 169.6 μg/mL L-NNP, respectively for 48 h). L-NNP reduced cell viability and increased ROS production and lipid peroxidation accompanied by a reduction in mitochondrial potential and GSH level in hepatoma cells (similar but much less significant changes were observed in normal cells). L-NNP resulted in a higher level of proapoptotic Bax and lower content of antiapoptotic Bcl-2 proteins. The cytotoxic properties of this compound were also confirmed in some in vivo studies performed on mice. Hepatic cancer bearing mice administered with high doses of L-NNP (40 mg/kg of body mass, daily for 7 days) lived longer, had a higher body mass and smaller volume of tumours compared with untreated and 5-fluorouracil treated animals (20 mg/kg of body mass). Additionally, a high dose of nitroxide resulted in a 126-fold higher level of ROS in the tumour tissue than in an untreated control. Simultaneously, it did not alter ROS level in the normal liver and kidney tissues (5-fluorouracil did not change ROS status in normal and tumour tissues). A high efficiency of L-NNP was explained by a significant increase in the oxidative stress in cancer cells, which they were not able to overcome (unlike normal cells) due to the low activity of antioxidative enzymes. Thus, nitroxide has proapoptotic properties and induces oxidative stress selectively in cancer cells [65].

An adamantyl nitroxide derivative (**18**) (Figure 5) revealed a higher (approx. 10-fold) anticancer activity against all the tested human hepatoma cells (Bel-7404 cells) than fluorouracil derivative (5-FU) used as a control drug. This nitroxide significantly inhibited tumour growth in a xenograft mouse model with low toxicity. Additionally, the adamantyl derivative suppressed the cell migration and invasion and induced the G2/M phase arrest and cell death. Further research revealed damage to mitochondria, increasing the generation of intracellular reactive oxygen species, cleavage of caspase-9 and caspase-3, and the activation of the apoptosis regulator proteins- Bax and Bcl-2 [66].

**Figure 6:**
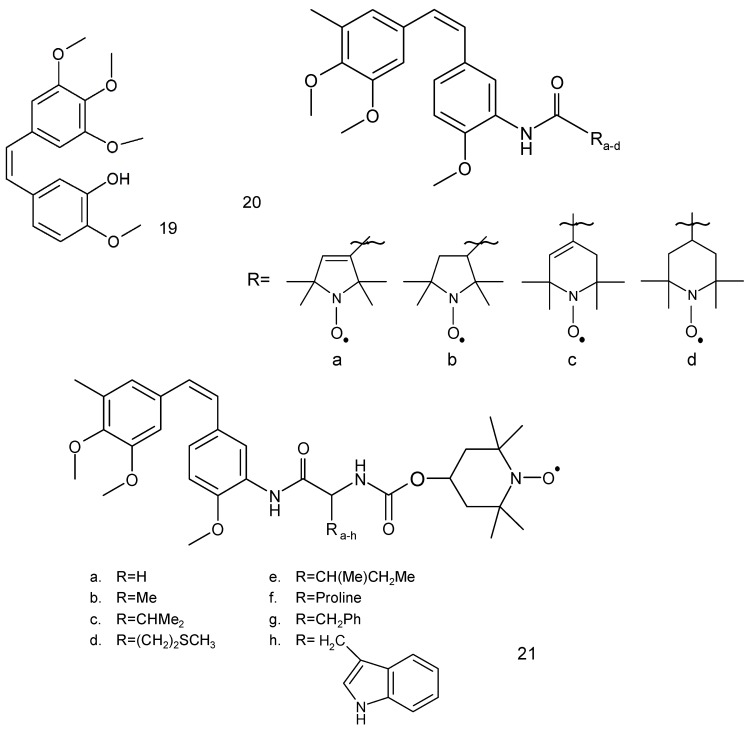
Natural and synthetic anticancer compounds: (**19**) combretastatin, (**20**) combretastatin with different spin label residues and (**21**) spin-labelled combretastatin derivatives.

Another anticancer drug, combretastatin A-4 (**19**) (Figure 6), is a natural phenol, which was first isolated from the bark of a South African tree *Combretum caffrum* by Pettit et al. [67]. This compound possesses a differential ability to cause vascular disruption in tumours and inhibition of tubulin polymerisation thus preventing cancer cells from producing microtubules.

Combretastatin A-4 derivative, which contains a 3′-*O*-substituted carbonic ether moiety, showed anticancer activities against four tumour cell lines, viz. MDA-MB-231, MCF-7, human leukaemia cell line (K562) and human lung cancer cells (A549 cells) with IC_50_ values in the range of 1 to 180 nM [68]. This compound effectively inhibited tubulin polymerisation to prevent mitosis in cancer cells, leading to G2/M cell cycle arrest and apoptosis. Combretastatin A-4 occurs in the form of two stereoisomers: *cis* and *trans*. The *cis* form exhibits better tubulin binding properties than the *trans* form.

Two series of spin-labelled combretastatin derivatives were synthesised and their cytotoxic activities were tested using four tumour cell lines (K562, SGC-7901, HeLa and HepG-2). The first group of spin-labelled combretastatin derivatives contained piperidine, pyrroline and pyrrolidine residues (**20 a–d**), while the second group included spin-labelled combretastatin and a piperidine derivative with different substituents (**21**) [69].

Spin-labelled combretastatin from first group compounds exhibited significantly less potent cytotoxicity against HepG-2 cell lines than the starting substance 3-amino-deoxycombretastatin A-4. While the nitroxides of the second group exhibited greater antitumor activity than etoposide, a clinically used anticancer drug, used as a reference compound.

### 3.3. Lung Cancer

**Figure 7:**
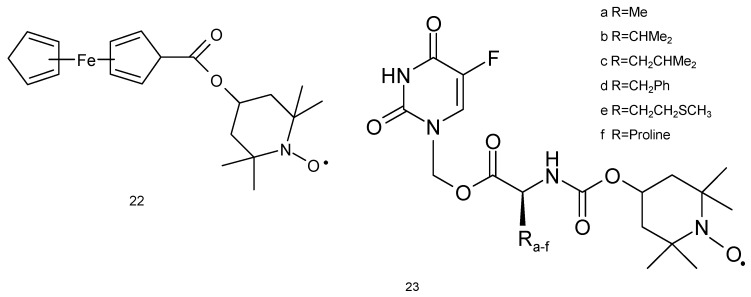
Nitroxides as anticancer agents: (**22**) FC-Tempo, (**23**) spin-labelled fluorouracil derivatives.

The studies of Wu and colleagues (2006) [70] revealed that metastatic lung cancer cell line 95-D is prone to FC-Tempo (4-ferrocenecarboxyl-2,2,6,6-tetramethylpiperidine-1-oxyl, (**22**) nitroxide therapy (Figure 7). This compound resulted in 50% reduction in the viability (390 μM, 48 h), while other nitroxide Tempol had no effect at the same concentration. FC-Tempo led to a significant release of extracellular LDH and enhanced apoptosis and caspase-3 activity (260 μM, 48 h). Other changes included altered cell cycle (G1 phase arrest) and enhanced activities of CAT and SOD. The cytotoxicity of FC-Tempo may result from the presence of ferrocenecarboxyl group at position 4, which may interact with cancer cell DNA or induce genotoxicity resulting from redox reactions [70].

About 20 novel spin-labelled camptothecin derivatives (**14**) have recently been reported and their antitumor activity in human non-small cell line (A549) has been established. Of the eighteen tested compounds, fourteen were characterised by a low IC_50_ (range IC _50_ value 0.045 to 0.090 μM) [61].

The toxicity of spin-labelled rotenone derivatives (**15**, **16**) was determined against A-549 cell line. All spin-labelled derivatives exhibited a cytotoxic activity against tested cancer cell lines, with IC_50_ values ranging from 0.471 to 0.738 mg/mL. However, spin-labelled rotenones showed significantly less toxicity than paclitaxel used as a reference compound [10].

Fluorouracyl is widely used for the treatment of many tumors such as colon and rectal cancer, breast cancer, gastrointestinal cancers (including anal, esophageal, pancreas and gastric cancers), head and neck cancer, basal cell cancer of the skin and actinic keratosis (squamous cell), neuroendocrine tumors, thymic cancers, cervical cancer, bladder cancer and hepatobiliary cancers. 5-FU was also used as a combination chemotherapy for lung cancer [71,72,73]. Six spin-labelled fluorouracil derivatives (**23**) were used as a cancer drug against A549 cell line (Figure 7). Two compounds (**23d** and **23f**) displayed anticancer activity at IC_50_ values of 2.76 and 2.38 μM, respectively, against lung cancer cells. The compound (**23f**) revealed approximately seven-fold more cytotoxicity against A-549 cell line in comparison to 5-FU [74].

### 3.4. Thyroid Cancer

The studies on TT and MZ-CRC-1 medullary thyroid carcinoma cell lines indicated that these cells are prone to Mito-CP_11_ [75]. It was reported to be active at very low concentrations (IC_50_ 0.38 and 0.89 μM for abovementioned cell lines, respectively (for 48 h) and triggered arrest of cell cycle in G0/G1 phase. The efficacy of nitroxide was compared with vandetanib—a drug commonly used in thyroid cancer therapy. Both compounds reduced cell viability at a similar level, but only Mito-CP_11_ induced PARP fragmentation and reduced expression of RET proto-oncogene. Therefore, both compounds act in different ways and the only nitroxide could induce apoptosis. These results were confirmed using some in vivo studies on mice, bearing induced medullary thyroid carcinoma. Additionally, nitroxide was reported to have an approximately equal level of anticancer activity of vandetanib, but Mito-CP_11_ reduced body mass of mice at a lower extent. Therefore, Mito-CP_11_ seems to be potent and less toxic than commonly used drugs [75].

Combretastatin A4 (**19**) is an anticancer drug used in treating patients with advanced anaplastic thyroid cancer. This compound possesses varying ability to cause vascular disruption in tumours and inhibition of tubulin polymerisation prevents cancer cells from producing microtubules.

Additionally, the spin-labelled analogues (**21a**,**b**) revealed the highest cytotoxicity against HepG-2, BGC-832 and HeLa cancer cell lines tested in comparison to 3-amino-deoxycombrethastin A-4 inhibitor of tubulin polymerisation and cytotoxic compounds.

### 3.5. Ovarian Cancer

**Figure 8:**
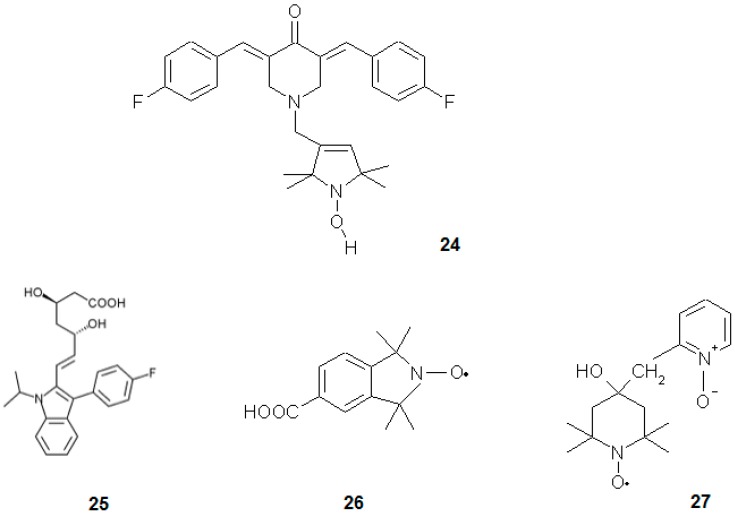
Nitroxides and other anticancer agents: (**24**) HO-3867, (**25**) fluvastatin, (**26**) CTMIO and (**27**) Tempicol-3.

Selvendiran et al. [76] investigated the influence of a novel group of compounds, diarylidenyl piperidones (DAPs), on ovarian cancer cell line (Figure 8). The most potent compound of this group was HO-3867 (**12**), which at 10 μM concentration (24 h) led to 80% reduction in the viability of A2780 human epithelial ovarian cancer cell line and slightly reduced the viability of normal human smooth muscle cells. It also increased oxidative stress and activity of caspase-3 accompanied by a drop of phosphorylation of STAT3 in cancerous cells. This compound acted differently in normal cells, being much less toxic and acting as antioxidant [76].

### 3.6. Lymphatic Cancer

Gariboldi et al. [2] investigated the influence of Tempol on HL-60 human leukaemia cell line. Nitroxide was found to be more toxic towards this cell line than normal bone marrow Detroit 6 cells (IC_50_ was 3 fold lower). This difference could be explained by the differential activity of Tempol in normal and cancerous cells, where it acted as antioxidant and pro-oxidant, respectively. In fact, it increased oxidative stress and upregulated p21^WAF1/CIP1^ in HL-60 cells. Additionally, Tempol triggered cell cycle arrest in G1 phase. The authors proved that p53 protein (which is not expressed in HL-60 cells) is not obligatory for cells to undergo oxidative stress-induced apoptosis and also the levels of Bax and Bcl-2 did not change significantly after Tempol treatment. Possibly, p21^WAF1/CIP1^ induced the p53-independent pathway of apoptosis [2]. In a subsequent paper [77], the authors proved that Tempol induces a decrease in cellular and mitochondrial GSH content. This nitroxide accumulated specifically in mitochondria, lowered mitochondrial membrane potential, oxygen consumption and ATP production. It also led to a decrease in the activities of complexes I, II and IV of the electron transport chain. The authors suggested two mechanisms of Tempol activity: one is the direct disruption of oxidative chain proteins leading to “electron leak” and ROS generation. The second mechanism was depletion of GSH that induced oxidative stress and led to apoptosis [77]. Nitroxides have also been found working as chemopreventives. For instance, Tempol was investigated in Atm^−/−^ mice, being especially prone to lymphoma development [78]. This type of rodents is characterised by an elevated level of oxidative stress, neurodegenerative alterations, high susceptibility to cancer and its early onset. Control (untreated) Atm^−/−^ mice lived for 30 weeks, while the animals administered with 10 mg of nitroxide per gram of food lived two-times longer. Tempol lowered oxidative stress in mice thymocytes, from both Atm^−/−^ and wild-type. In the first type of mice, nitroxide prevented further decrease in the mitochondrial potential and lowered the level of oxidative stress marker haemo-oxygenase-1. The authors proposed a chemopreventive action affected by the antioxidant properties of nitroxide. They also observed that Tempol caused a notably slower gain of body mass and slower proliferation rate of thymocytes in Atm^−/−^ mice. In combination with the slower proliferation of splenocytes under in vitro conditions, nitroxides also influence the signalling pathways connected with cell proliferation [78]. Atm^−/−^ mice were also used as a model in other studies, devoted to CTMIO (**26**) nitroxide. In this case, a stronger chemoprevention in the Atm^−/−^ mice was reported [7].

The oxidative mechanism of action of Tempo nitroxide was observed in mouse lymphoma cell line (L5178Y). This nitroxide induced a time- and concentration-dependent intracellular production of ROS and depletion of glutathione. It also increased caspase-3 and 7 activities, decreased expression of anti-apoptotic proteins including Bcl-2, Bcl-xL and McL-1 and consequently led to apoptosis [79].

### 3.7. Other Cancers

Another nitroxide that might find application in chemotherapy is Tempicol-3 (**27**). It was observed to be cytotoxic towards Yoshida’s sarcoma, both in vivo in rats and in vitro in mice cell lines of the neoplastic phenotype. It was a suppressor of tumorigenesis and inducer of apoptosis in cancerous cells while being nontoxic to the normal cells [80].

Recently we have shown that Pirolin could enhance the pro-oxidative effect of docetaxel (DTX) and increase TNF-α expression in the brain tissue when administrated in rats with doxorubicin (DOX), paclitaxel (PXL) and docetaxel. On the other hand, Pirolin decreased DOX-induced oxidative damage and protected DNA against DOX- and PTX-induced damage. PL reduced PARP-1 cleavage and nNOS expression evoked by DOX and DTX. We found nitroxides as the new cytoprotectants of the central nervous system against DOX-, DTX- and PTX-induced oxidative stress [81].

Both Mito-CP and Mito-CP-Ac exhibited similar dose- and time-dependent antiproliferative effects when tested on human pancreatic (MiaPaCa-2 and Panc-1), human epidermoid carcinoma (A431) and bladder (253J) cancer cells [57].

The studies conducted on the human melanoma cell lines (SK-MEL28, A375 and RPMI-7951) showed that Mito-CP effectively induced oxidative stress and in consequence depolarization of the mitochondrial membrane and apoptosis. Additionally, these tests exhibited a greater efficacy of nitroxide in comparison to a proto-oncogene BRAF inhibitor, Vemurafenib (PLX4032) [82].

It was shown that the redox nanoparticles (RNPN) including curcumin and Tempol induced strong apoptosis in the prostate cancer (PC-3) cells compared to free curcumin. While, the intravenous injection of RNPN to old nude mice (BALB/C) suppressed tumour growth in vivo, which was due to the increased bio-availability and significant ROS scavenging at the tumour sites [83].

The previously mentioned spin-labelled rotenone derivatives (**15**, **16**) were used against DU-145, Kb and Kbvin cell lines. Rotenone revealed anticancer activity by the induction of apoptosis via inhibiting the microtubule assembly [84]. The spin-labelled analogues of rotenone exhibited cytotoxicity against all tested cells lines. Interestingly, all the compounds revealed higher activity than paclitaxel against KBvin cells line and nitroxides 16a and 16d were the most cytotoxic against this cell line (IC_50_ 0.075 and 0.092 μg/mL, respectively) [10].

The spin-labelled derivatives of combretastatin A-4 (**21a**–**c**) exhibited significant cytotoxicity against human leukemia cell line K562, gastric cancer cell line SGC-7901 and HeLa cancer cells and were more active than etoposide, an antineoplastic drug [69].

Nitroxides (**21a–c**) revealed more cytotoxicity against two cell lines, human gastric cancer (BGC-823) and HeLa cancer cell lines than 3-amino-deoxycombrethastin A-4, which is an inhibitor of tubulin polymerisation and a cytotoxic compound.

Combretastatin A-4 inhibited cell growth and metastasis in bladder cancer cells. It was applied in combinations of carboplatin and paclitaxel in a variety of tumour types, including ovarian cancer, small-cell lung cancer, adenocarcinoma of the oesophagus-gastric junction and malignant melanoma [85]. The high effectiveness in ovarian cancer treatment demonstrated that this disease could be cured by employing vascular strategies. It was observed that the application of spin labelled analogues can improve its effectiveness.

Some spin-labelled combretastatin derivatives used against HepG-2 cell lines were also tested against K562, SG7901 and HeLa cell lines. Compounds (**20a**–**d**) revealed lower cytotoxicity against all cell lines than 3-amino-deoxycombretastatin A-4. On the other hand nitroxides (**21a**–**c**) exhibited the greatest cytotoxicity against all cancer cell lines than the first group. The mechanistic analysis showed that this compound (21a) can effectively inhibit tubulin polymerisation to prevent mitosis in cancer cells, leading to cell cycle arrest and apoptotic cell death better than the etoposide reference drug [69].

Some spin-labelled fluorouracil derivatives were tested as anticancer compounds against prostate cancer cell line (DU-145) as well as parental Kb and multidrug-resistant Kbvin cell lines. However, these studies revealed little lower anticancer activity of spin-labelled compounds than fluorouracil [74].

The previously described camptothecin and its spin-labelled analogues were also tested on Kb and Kbvin cell lines. Spin-labelled derivatives (**14a**) and (**14b**) showed the highest cytotoxicity against the Kbvin cell line IC_50_ (0.057 and 0.072 μM, respectively).

**Figure 9:**
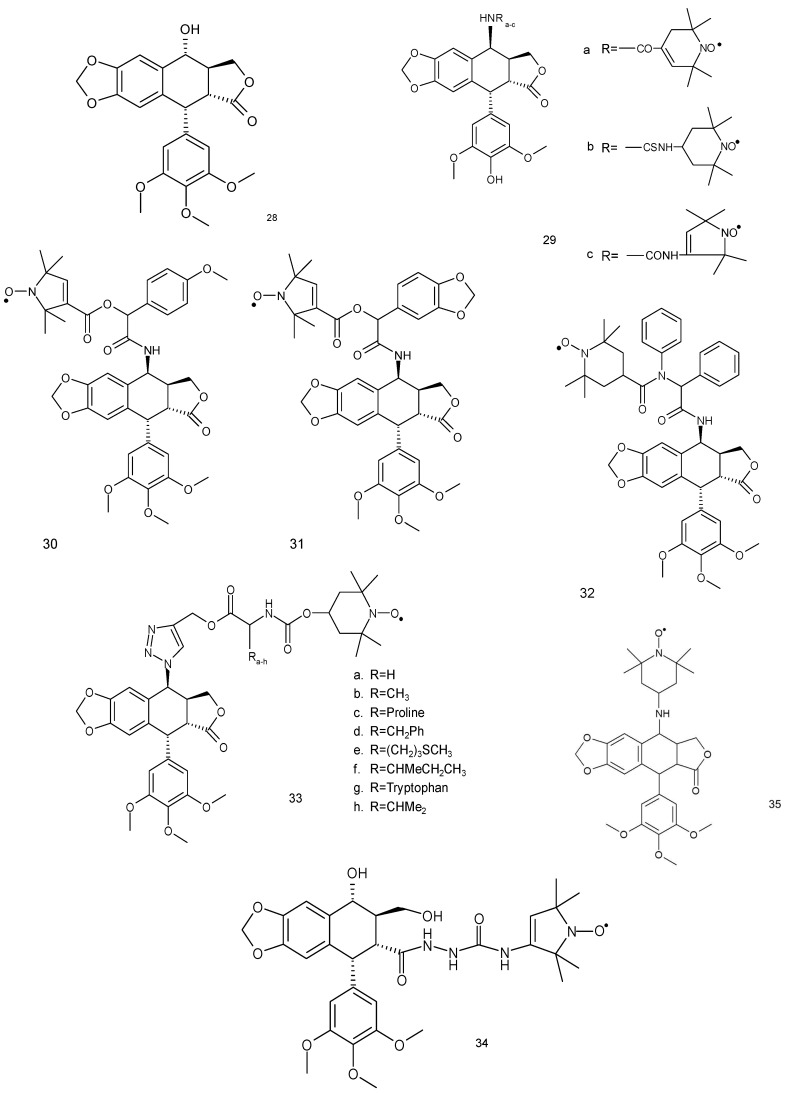
Podophyllotoxin and its spin-labelled derivatives: (**28**) podophyllotoxin, (**29**–**35**) spin-labelled podophyllotoxin derivatives.

The podophyllotoxin derivatives are natural compounds belonging to lignan family. They have been characterised for their antineoplastic and antiviral properties (Figure 9). The semisynthetic derivatives of podophyllotoxin, such as etoposide and teniposide, are widely used in chemotherapy of various tumours. The mechanism of action of podophyllotoxin is related to the inhibition of microtubule assembly. However, the toxicity of etoposide and teniposide is associated with the interaction with DNA and inhibition of topoisomerase II [86].

The spin-labelled podophyllotoxin derivatives (**29a**–**c**) exhibited antitumor activity against human nasopharyngeal carcinoma Kb, lung cancer AS49 and stomach carcinoma SGC-7901 cells, mouse leukaemia L12 10 and P388 cells. Nitroxides (**29a**–**c**) displayed comparable or superior activity to etoposide for the inhibition of these cell lines. The cell line p388 showed the highest sensitivity for the used compounds [87].

Spin-labelled podophyllotoxin derivatives exhibited better antitumor activity than podophyllotoxin. Three spin-labelled podophyllotoxins analogues were tested against four human cancer cell lines (A-549, DU-145, Kb and Kbvin). Both compounds, (**30** and **31**), exhibited better cytotoxic activity against all cell lines in comparison to etoposide used as a reference anticancer drug. Nitroxides (**30**) showed a higher cytotoxic activity against Kbvin cells line than etoposide [88].

Using the same cell lines, Yang and colleagues (2014) [74], used another series of spin-labelled podophyllotoxin derivatives (**33** and **34**). These compounds displayed a cytotoxic activity against the tested cancer cell lines (A-549, DU145 and Kb); however, the IC_50_ value was higher in comparison to etoposide. On the other hand, nitroxide after 33 h showed a higher cytotoxic activity than etoposide (IC_50_ 6.30 to > 10 μM) against Kbvin cell line.

Another spin-labelled ester derivative of podophyllotoxin was used against neoplastic cell lines (K562, HL-60, SPCA-1, Lewis and L-1210). Most of these compounds showed cytotoxicity against all cancer cell lines, except for SPCA-1 [89].

Another spin labelled derivative of podophyllotoxin (**35**) was tested against leukaemia and osteosarcoma cell lines. This compound can arrest cycle in the S phase and induce apoptosis by the activation of caspases-3, 8 and 9. In a DNA fragmentation experiment, it also caused DNA laddering pattern, further confirming that it can induce apoptosis. The release of cytochrome-c and the increase of proapoptotic proteins Bax and Bak also indicated apoptosis through mitochondrial pathway [90].

### 3.8. Nitroxides and Cis-platin Toxicity

**Figure 10:**
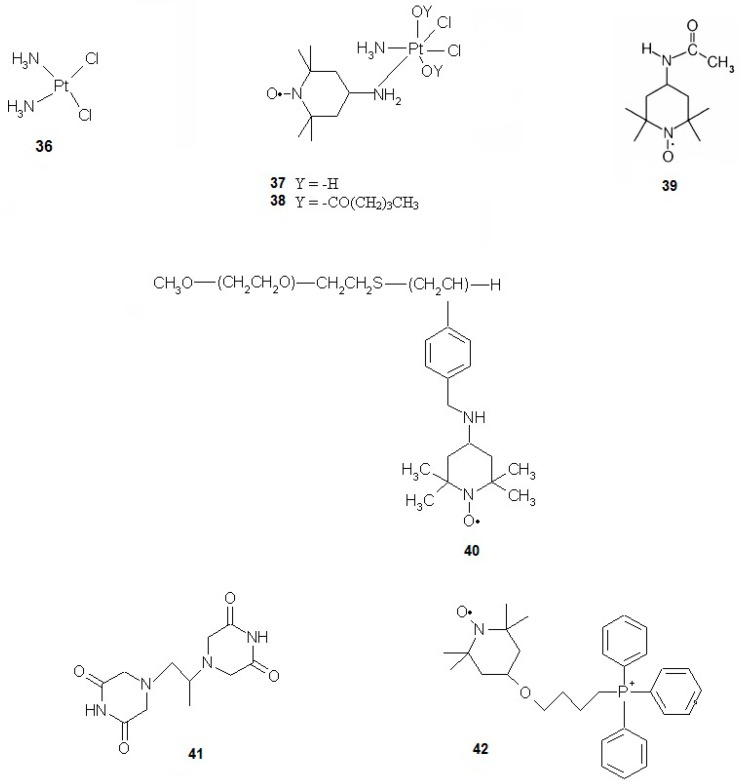
Spin-labelled *cis*-platin derivatives and other anticancer agents: (**36**) *cis*-platin, (**37**) Nx-Pt I, (**38**) Nx-Pt II, (**39**) Tempace, (**40**) PEG-b-PMNT, (**41**) dexrazoxane and (**42**) Mito-Tempol-4.

*Cis*-platin (**36**) is one of the most commonly used chemotherapeutic agents (Figure 10). It has been used for decades and currently some derivatives of cisplatin have been developed in order to reduce its toxicity to normal cells. Such an example is a complex of *cis*-platin and nitroxide, namely, Nx-Pt II (**38**), which led to cell cycle arrest and induced apoptosis in HeLa human cervical carcinoma and H1299 human lung carcinoma cell lines [91]. Some in vivo analyses performed on mice revealed that some complexes, e.g., Nx-Pt I (**37**), induce cancer resistance against a drug at a 2.5-fold slower rate than *cis*-platin. When used in combination for the treatment of leukaemia, *cis*-platin and Nx-Pt I (0.6 and 1.4 mg/kg of body mass, respectively) showed a synergistic effect. After 60 days of the treatment, 100% of mice were cured, while the drugs administered alone showed no effect as almost all mice were dead. In case of nitroxide-platin complex, the slower development of resistance and mentioned synergism suggested that anticancer mechanism of the complex is different from that of *cis*-platin alone. These complexes possess antioxidative properties that alleviate side effects, such as nephro- or neurotoxicity, which results in the higher viability of animals and efficacy of therapy [91].

### 3.9. Nitroxides and Doxorubicin Toxicity

Doxorubicin (DOX) belongs to the anthracycline antibiotics group that is commonly used in anticancer therapy of many tumours. However, it is also toxic towards normal cells, especially those of heart, liver and kidneys. This adverse action is related to the conversion of DOX to semichinone radical, which reacts with DNA and/or oxygen resulting in the generation of the superoxide anion radical. The latter, after dismutation to hydrogen peroxide takes part in the Fenton reaction, which yields hydroxyl radical. It induces oxidative damage to lipids, proteins and DNA of many cells, especially those with a low antioxidant pool such as cardiomyocytes. The application of nitroxides may alleviate side effects of DOX, protect normal cells from oxidative stress and enhance the cytotoxic activity of DOX against cancer cells [14,31,92].

Czepas et al. (2008) [31] evaluated the potential of four nitroxides, with see Tempo, Tempol, Tempamine and Tempace (**39**), in reducing the toxicity induced by DOX in normal cells. These derivatives differed in substituent at position 4 of the piperidine ring. The experiments carried out on B14 immortalised hamster fibroblast cell line revealed that the most effective compound in alleviating the side effects of DOX was Tempace. Similarly, to Tempo and Tempol, it increased the viability of cells unlike Tempamine, which at higher concentrations (500–2000 μM) acted as a pro-oxidant and enhanced the toxic effect of DOX. The effect of the compounds on lipid peroxidation induced by DOX was also evaluated. Again, Tempamine was the only nitroxide that slightly increased damage to lipids, while the three other nitroxides had protective properties. EPR studies revealed that the bioreduction of nitroxides may be significant for their protective activity. The lowest rate of reduction was noted for Tempace, which was also the most efficient antioxidant to protect from DOX-induced toxicity. The least efficient Tempamine was reduced much faster. It has been confirmed that the type of substituent at position 4 of the piperidine ring is crucial for the activity of nitroxides [31]. Antioxidant properties of hydroxylamines were also confirmed; however, they were less efficient than nitroxides [89].

Yoshitomi et al. [92] investigated the activity of RNP^N^ in alleviating DOX-related side effects. This molecule consists of an ethylene glycol copolymer and aminomethylstyrene (PEG-*b*-PMNT) (**40**) with attached nitroxide. These macromolecules are pH-sensitive and preferentially accumulate at inflamed sites, for instance, the cancer environment. The microenvironment of a cancer site is characterised by a lowered pH due to hypoxic conditions and enhanced glycolysis (Warburg effect). Under such conditions, polymer releases Tempo that interacts with cancerous cells. ROS generated by DOX lead to activation of NF-κB that promotes cancer progression and drug resistance. The antioxidant effect of Tempol reduces the level of DOX-derived ROS and prevents the development of resistance towards drugs in cancer cells. The authors showed that in comparison with free Tempol, RNP^N^ circulated longer in the blood of mice, accumulated at cancer site at a high level and significantly reduced the activity of NF-κB in the nucleus of colon-26 cancer cells. The administration of RNP^N^ resulted in a significant drop of ROS and TNF-α levels when compared to free Tempol and the untreated controls. The subsequent administration of DOX few days after RNP^N^ resulted in an approximately 6-fold reduction in tumour volume in mice, while DOX alone yielded much less evident effect. The influence of RNP^N^ on DOX-induced toxicity towards normal cell was also evaluated. The administration of RNP^N^ (unlike Tempol) significantly reduced the activity of biomarkers of cellular damage CPK and LDH and reduced the level of ROS and lipid peroxidation in cardiomyocytes of mice. In summary, RNP^N^ reduced the level of inflammatory cytokines, production of ROS and cardiotoxicity, while simultaneously enhanced the anticancer activity of DOX. It was more potent than free Tempol, which is administered intravenously and transported through the whole body, does not act as precise as RNP^N^ when specifically released in the microenvironment of a tumour [92].

Dickey and colleagues (2013) [14] employed SHR rats bearing implanted SST-2 mammary cancer cells to investigate the toxicity of DOX. They attempted to reduce the toxicity by using nitroxide Mito-T-4 [Mito-Tempol] (**42**). Dexrazoxane (**41**), which is used in DOX therapy to prevent oxidative stress and alleviate chemotherapy-related side effects, was used as the reference. The analysis of mitochondrial fractions of heart and cancer cells of rats revealed that Mito-T-4 accumulated strongly in mitochondria of cardiomyocytes than cancer cells. While DOX and Mito-T-4 combined therapy led to a significant reduction in the body mass of the rats (comparing to control and single DOX), it alleviated some cardiotoxicity parameters such as plasma glucose level and changed lymphocyte and granulocyte counts. Dexrazoxane combined with DOX, on the other hand, alleviated the reduced levels of plasma albumin, alanine-1-transferase, bilirubin, glucose and lipids. In order to assess cardiotoxicity, the damage to cardiomyocytes was also evaluated. The high extent of damage induced by DOX was lowered by the co-administration of Mito-T-4, but it was slightly less efficient than dexrazoxane. The relevant difference between Mito-T-4 and dexrazoxane was that only nitroxide enhanced DOX toxicity against cancer cells. Both alleviators inhibited apoptosis and the induced autophagic protective pathway, thus protecting the heart of rats and reducing the oxidative damage to DNA induced by DOX in this organ. Therefore, Mito-T-4 might be used in DOX therapy to increase its anticancer activity and protect the heart from its adverse effects [14].

Recently twelve new spin-labelled conjugates of known antioxidants trolox (TroH), trolox succinate (TroS), α-tocopheryl succinate (α-TOS) have been reported and compared with their unlabelled counterparts. TroS inhibited the growth of human cancer cells: myeloma, mammary adenocarcinoma, hepatocarcinoma, T cells leukaemia, histiocytic lymphoma and T-cellular leucosis in an IC_50_ range of 24 μM to ≥300 μM. In contrast nitroxide derivatives, viz. TroH, TroS and α-TOS, could not inhibit the proliferation of any cancer cells [93].

## 4. Nitroxides in Aging and Diseases

One of the theories of aging is Harman’s theory of free radicals [94,95,96]. It hypothesizes that the aging process occurs at the molecular level as a consequence of the oxidation of vital macromolecules by reactive oxygen species (ROS) generated in the cells and tissues. ROS are generated mainly by mitochondria (85–90% of cellular ROS) during aerobic cellular metabolism under normal physiological conditions [97,98]. About 2–3% of the total oxygen consumed in mitochondria, considering scavenging, is converted to ROS [97]. However, the weakened antioxidant system causes an increase in the ROS value from 0.25% to 11% depending on the organism and respiratory rate [99]. The reactive oxygen species, such as superoxide anion radicals, hydrogen peroxide, hydroxyl radicals are released within the mitochondria from electron transport chain. These products and other reactive species such as singlet oxygen, hypochlorous acid, and nitric oxide can be generated during oxidative burst of phagocytic cells [100,101].

The ROS can be generated from exogenous sources like air and water pollution, ionizing and ultraviolet radiations, tobacco smoke, cooking (smoked meat, frying in vegetable oil), drugs (halothane, paracetamol, bleomycin, and doxorubicin), transition metals (highly toxic: Cd, Hg, Pb, As and less toxic Fe, Cu, Co, Cr), pesticides, organic solvents hypoxia (ischemia and reperfusion), hyperoxia, acute exercise, etc. [102,103,104]. Aging causes decrease in the ability to cope with environmental stress resulting in increased susceptibility and vulnerability to diseases.

However, in homeostasis, there is a counterbalance between ROS generation and its elimination. The disruption of equilibrium between ROS formation and its removal leads to oxidative stress and associated diseases. The disorder may be due to overproduction of reactive oxygen species or degradation of the antioxidant system or both. Moreover, each cell also has a suitable repair system to repair defective proteins, lipids, and DNA. However, the scope of these systems is limited and may be damaged by ROS. As age increases, the activity of antioxidant and repair systems decreases as the release of reactive oxygen species increases.

Aging is a result of increased intracellular oxidative stress and chronic inflammation, which leads to the damage of nucleic acids, proteins and lipids [105]. A higher lipid peroxide serum level was found in older subjects than in younger ones irrespective of gender. However, no difference was found between sexes by age group [106]. A further decreased level of total antioxidant and antioxidant enzyme activities (e.g., superoxide dismutase and glutathione peroxidase) were observed [107]. At the same time, the oxidative DNA damage biomarkers of 8-oxo-7,8-dihydro-2′-deoxyguanosine (8-oxodG) and 8-oxo-7,8-dihydroguanine (8-oxoGua) were observed to change with age [108]. Aging also shows significantly higher levels of lipofuscin (“aging pigment”), a product of oxidative damage to cellular membranes. The most authentic lipofuscin is a complex mixture of oxidized protein, lipid degradation residues, minor amounts of carbohydrates and transition metals such as iron, copper, zinc, and other [109].

According to the mitochondrial theory of aging mitochondria act as the primary sources as well as the primary targets of ROS. The oxidative stress in mitochondria plays an important role in aging and health status of many vital organs [110]. A decreased antioxidant enzyme activity in living cells leads to increased risk of oxidative damage to important molecules like DNA, RNA, proteins, enzymes, lipids, and carbohydrates. There are three isoforms of superoxide dismutase: SOD1 (Cu, Zn SOD) located in mitochondrial intermembrane space and cytosol, SOD2 (Mn SOD) located in mitochondrial matrix and SOD3 (Cu, Zn SOD) located in extracellular space [111]. The low molecular weight antioxidants such as glutathione, ascorbic acid, α-tocopherol, carotenes, lipoic, and uric acids also play an important role in scavenging free radicals [112]. All SOD isoforms suppress the superoxide anion radical (O_2_^−^^•^), precursor of other oxygen reactive species. The dismutation of O_2_^−^^•^ by SOD leads to the generation of a very toxic agent to the cell, namely, hydrogen peroxide. However, hydrogen peroxide is rapidly eliminated by glutathione and thioredoxin in the mitochondrial matrix and other enzymes such as catalase and glutathione peroxidase [99,113,114].

The high levels of ROS are detrimental to cells, leading to the onset of aging and many acute and chronic aging-related diseases such as hypertension, atherosclerosis, coronary heart disease, cancer, metabolic syndrome, diabetes mellitus, chronic pulmonary disease, cataracts, age-related macular degeneration, myocardial infarction, cerebrovascular disease, sepsis, rheumatoid arthritis, and neurodegenerative disease (Alzheimer’s and Parkinson’s diseases) [102,115].

However, a moderate level of oxidative stress may induce and modulate an adaptive cellular response, which can be beneficial for redox signaling and cell survival [116]. The antiaging action is correlated with the attenuation of oxidative damage, modulation of glycemia and insulinemia, hormesis, and caloric restriction [105,117]. Physical exercise, especially aerobic training, may be proposed as an effective intervention in the prevention and treatment of hypertension and cardiovascular disease by reducing oxidative stress [118].

Nitroxides may also be used as antioxidants to delay aging especially for those having accelerated oxidative stress due to overweight, obesity leading to diabetes mellitus, inflammation, cardiovascular disease, and others.

Our previous paper showed that nitroxide protects biological materials from the effects of ionizing radiation. Nitroxide protects the healthy cells but not the cancerous ones from ionizing radiation. The differential effect of nitroxides in both cells correlates with the oxidative state in the tumor tissue as opposed to the reducing status of normal cells [119]. Nitroxide also showed to have protective effects against UV and visible (VIS) radiation-induced photo-aging of the skin cells. In addition, preliminary studies showed its higher efficacy as UV-VIS filters than conventionally used vitamins C and E [119].

Tempol and its diamagnetic derivatives (hydroxylamines) are widely used in in vivo and in vitro studies. The application of Tempol as a protective agent against oxidative stress in biomedical studies has extensively been described by Wilcox [16]. Tempol therapy in diabetic rats significantly improved the changes in the extracellular matrix of the heart. In addition, the levels of TGF-β, ROS or serum LDH, CK-MB and MMP-2 activity were improved and maintained the integrity of cardiac tissues in diabetic rats [120].

In the fat-fed models of metabolic syndrome, Tempol was found to prevent left ventricular hypertrophy and heart failure [121]. Tempol was also used to protect against ischemia/reperfusion injury in many organs, including the heart, brain, kidney, and ovary [122,123,124,125]. Tempol also improves blood pressure and endothelial function [17,126,127].

Caloric restriction is known to prolong life [128]. It was reported that Tempol significantly extends the survival of mice and the animals were found to continue an active life even after aging [129]. Obesity is associated with many diseases such as hypertension, cardiovascular disease, type 2 diabetes, and carcinogenesis. Samuni and colleagues (2010) showed that adding Tempol to the mice diet prevents obesity by suppressing adipogenesis causing significant weight loss without toxicity [130].

Tempol showed a profound effect on body weight, atherosclerosis, hyperlipidemia, and inflammation in established models of obesity-associated hyperlipidemia and atherosclerosis [131].

In older mice, plasma lipid peroxidation and C-reactive protein were significantly increased as compared to younger ones, which were counteracted by an oral dose of Tempol.

The Tempol therapy, which leads to a reduction in mortality, is based on the alleviation of chronic inflammation and improvement of the immune system function [132].

Tempol mitigated renal damage in animal models of hypertension associated with obesity [133,134]. Tempol therapy reduced renal oxidative stress, improved endothelial function, and reduced glomerular damage in a model of obesity and hypertension [134].

The administration of Tempol significantly reduced the progressive sclerotic and proliferative glomerular changes in hypertensive rats and prevented the occurrence of hypertension, increased urinary excretion of protein, and lipid peroxidation [135,136]. In addition, the treatment with nitroxide normalized the activity of ERK1/2, JNK, and BMK1 [136]. The protective effects of Tempol were found to be linked to the mitogen-activated protein kinase signaling pathway as a target for tissue protection.

It was reported that Tempol can remove ROS and attenuate the inflammation and tissue damage associated with carrageenan-induced pleurisy in rats [137].

Nitroxides can be used as a preventative or pharmaceutical drug against age-related macular degeneration (AMD) and cardiovascular disease.

**Figure 11:**
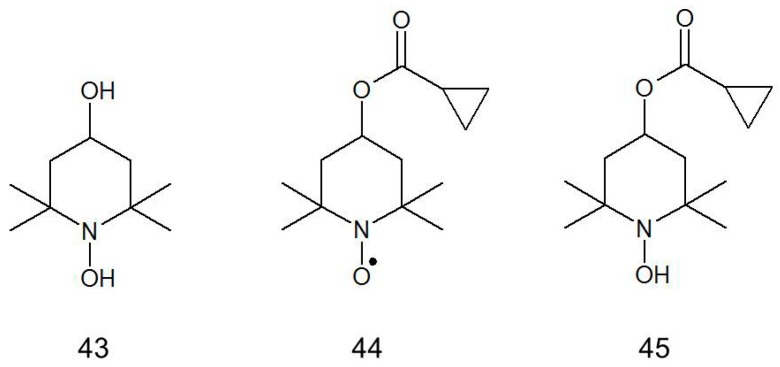
Nitroxides and hydroxylamines used in age-related macular degeneration.

Tempol and its derivatives, e.g., Tempol-hydroxylamine (Tempol-H) (**43**), ester of cyclopropanecarboxylic acid (**44**), and Tempol-H hydroxylamine (OT-551) (**45**) were used as preventive and pharmaceutical drugs in age-related macular degeneration (AMD) [138] (Figure 11).

Nitroxide and its hydroxylamine derivatives have demonstrated anti-inflammatory, antioxidant and anti-angiogenic activities in prophylactic and therapeutic vascular dysfunctions in patients including chronic smoking patients. AMD is the result of normal aging processes and pathology. Apart from aging, faster AMD is a consequence of unhealthy life style i.e., died, smoking, alcohol consumption, lack of movement, and environmental factors. Inhibition of this process is possible by excluding the accelerating factors as well as the introduction into the diet antioxidants such as ascorbic acid and α-tocopherol, metals (Zn, Cu), and omega-3-polyunsaturated fatty acids [139].

The early etiology of AMD involves the degradation of retinal pigment epithelial (RPE) cells, which are sensitive to oxidative stress [140]. Tempol-H has strong protective effect against oxidative damage in the eye. Furthermore, it suppressed the photo-oxidative processes initiated by the RPE lipofuscin fluorophore bis-retinoid (A2E) and quenched singlet oxygen more effectively than Trolox and α-tocopherol [141].

I was shown in young adult smokers that Tempol effectively reversed the damage of impaired endothelial function. This effect was caused by the inhibition of oxidative stress and increased bioavailability of nitric oxide [142,143].

Recently, Tempol has been shown to relieve and prevent paclitaxel-induced neuropathic pain in rats by reducing the levels of inflammatory cytokines and free radicals in dorsal root ganglia [144].

## 5. Concluding Remarks

Nitroxides are the promising candidates of cancer therapy. Several studies have confirmed their efficacy against breast, liver, lung, thyroid, ovarian and lymphatic cancers. Their anticancer effects have been established by reducing the viability of tumor cell lines, slowing down ATP production, damage to electron transport chain, mitochondrial mitosis, induction of apoptosis (by regulating levels of pro- and anti-apoptotic proteins), cell cycle arrest in G1 phase, strong RFT production and the induction of oxidative stress in cancer cells and tumor tissue. The effect of nitroxides is highly precise and selective; meanwhile, in normal cells, they exhibit completely different activity. Generally, they act as protectants and antioxidants slightly affecting the viability of healthy cells. They protect against weight loss and in some cases effectively prevent the onset of cancer and prolong the life of the experimental animals.

Several reports have documented the synergistic effects of nitroxide with anticancer drugs, for example, Mito-CP_11_ with fluvastatin in breast cancer and with 2-DG in liver cancer. Nitroxides were also found to be highly effective against thyroid cancer. It has also been shown that nitroxides may be more effective than commonly used antineoplastic drugs such as 5-fluorouracil, vandetanib and many others. Nitroxides decreased the toxicity in Chinese hamster cells (line B14), mouse cardiomyocytes and SHR rats, being treated with doxorubicin. Their protective effect was associated with a decrease in oxidative stress and the consequent inhibition of oxidation of proteins and lipids, reduction in cardiac damage and inhibition of apoptosis in this organ. On the other hand, nitroxides when combined with a drug intensify the oxidative stress in the malignant cancer cells.

Nitroxides, ligated to *cis*-platin derivatives, exhibited in vitro toxicity against several tumour cells and when these complexes were co-administered with *cis*-platinum under in vivo experiments, a high therapeutic value and significantly slower development of drug resistance were observed.

Thus, it can be said that spin-labelled analogues of natural antineoplastic compounds could be a better choice as an antitumour agent than their precursors. Anti-inflammatory, angiogenic and antioxidant properties of nitroxides give the opportunity to use them as effective drugs and antioxidants to protect the cells and tissues from oxidative stress.

## Abbreviations

BMK1	Big mitogen-*activated* protein kinase 1
CAT	catalase
CK-MB	creatine kinase-MB
ERK1/2	extracellular signal-regulated kinase 1 and 2, fluorouracil (5-FU)
GSH	glutathione (reduced form)
JNK	c-Jun N-terminal kinase,
LDH	lactic dehydrogenase
MMP-2	matrix metalloproteinase-2
NADH	(nicotinamide adenine dinucleotide, reduced form)
PARP	(poly(ADP-ribose) polymerase)
SOD	superoxide dismutase
